# The Age of Older Patients Requiring Prolonged Mechanical Ventilation Is Not the Sole Determinant of Their Long-Term Survival

**DOI:** 10.3390/medicina60020211

**Published:** 2024-01-26

**Authors:** Chienhsiu Huang

**Affiliations:** Division of Chest Medicine, Department of Internal Medicine, Dalin Tzu Chi Hospital, Buddhist Tzu Chi Medical Foundation, No. 2, Min-Sheng Road, Dalin, Chiayi 62247, Taiwan; hgssport@yahoo.com.tw or dm550671@tzuchi.com.tw; Tel.: +886-9-2155-2418

**Keywords:** old age, prolonged mechanical ventilation, discharge outcomes, weaning outcomes, long-term survival

## Abstract

*Background and Objectives:* There are few data on the effects of prolonged mechanical ventilation on elderly patients. Our objective is to investigate the effects of prolonged mechanical ventilation on older patients’ successful weaning and long-term survival. *Methods:* We examined how aging affected the course and results of elderly patients on prolonged mechanical ventilation by contrasting five age groups. Age, sex, cause of acute respiratory failure, comorbidities, discharge status, weaning status, and long-term survival outcomes were among the information we gathered. *Results:* Patients on prolonged mechanical ventilation who had undergone tracheostomy and had been successfully weaned had a greater one-year survival rate. The 1-year survival rate was poorer for older patients with four or more comorbidities. Regarding the 5-year survival rate, the risk of death was 45% lower in the successfully weaned patients than in the unsuccessfully weaned patients. The risk of death was 46% lower in patients undergoing tracheostomy than in those not undergoing tracheostomy. Older prolonged mechanical ventilation (PMV) patients with four or more comorbidities had an increased risk of death. *Conclusions:* When it comes to elderly patients on prolonged mechanical ventilation, there are other factors in addition to age that influence long-term survival. Long-term survival is likewise linked to successful weaning and undergoing tracheostomy.

## 1. Introduction

Patients on prolonged mechanical ventilation (PMV) are defined as those who must use a mechanical ventilator for at least 6 h daily for at least 21 consecutive days [1]. The National Public Health Insurance Ventilator-Dependent Patients Comprehensive Care System began in Taiwan in July 2000 [2]. This program covers mechanical ventilator care in the following settings: the intensive care unit, the respiratory care center (RCC; a subacute stage for patients receiving ventilator support for >21 days), the respiratory care ward, and the home care service. Patients who fulfilled the standards of the Taiwan National Health Insurance were eligible for admission to the RCC. If a patient fulfilled the conditions of the Taiwan National Health Insurance, they might be admitted to the RCC [3].

Society is aging worldwide, and most developed countries have accepted the age of 65 years as a cut-off to define older individuals. Due to the increase in aging populations and the proportion of patients with multiple comorbidities, there is an increasing number of older patients who suffer from acute respiratory failure and require PMV. Aboussouan et al. showed that patients aged ≥65 years account for 78% of patients requiring PMV [4]. Similarly, a study conducted in Taiwan showed that patients aged ≥65 years account for 76.6% of patients requiring PMV [5]. Information about the effects of PMV on the outcomes and progress of older patients has been limited. The present study aimed to investigate the effects of older age (age ≥65 years) on successful weaning, ventilator dependence, RCC mortality, general ward mortality, comorbidity, and long-term survival during a 6-year period and identify risk factors associated with long-term survival in older patients requiring PMV.

## 2. Materials and Methods

### 2.1. Setting

This study was carried out at the tertiary general hospital Dalin Tzu Chi Hospital in Chia-Yi, Taiwan. Patients requiring PMV are taken care of by our RCC, an acute-care hospital weaning facility.

### 2.2. Study Design

The medical records of 574 patients who were hospitalized at the RCC between January 2012 and December 2017 were examined retrospectively. This 6-year study retrospectively explored the influence of increasing age on the outcomes and progress of older patients requiring PMV by comparing the following five age cohorts: patients aged 65–69, 70–74, 75–79, 80–84, and ≥85 years. The researchers calculated the survival of all participants from the date they were admitted to the RCC until their death or 31 December 2018. These were the conditions for exclusion: (1) Patients are considered lost follow-up patients if they were discharged from the hospital without an outpatient visit medical record. (2) We are unsure of the patient’s survival until 31 December 2018, even if the patient has a medical record of their outpatient appointment after being discharged from the hospital. We collected data on their age, sex, comorbidities, causes of respiratory failure, RCC discharge status, weaning status, survival time, and long-term survival rate. Data were extracted retrospectively for all participants from their medical records. Survival curves for all older patients requiring PMV, those weaned successfully, and those weaned unsuccessfully were compared separately based on five age cohorts. We also examined the differences in age, presence of other medical conditions, causes for respiratory failure, discharge status from the RCC, whether or not the patient received a tracheostomy, progress in weaning off mechanical ventilation, length of survival, and 1-year survival rates between patients over 65 years old and those under 65 years old who required PMV.

### 2.3. Definitions

We defined successful weaning as patients being able to breathe independently without the use of a mechanical ventilator for five consecutive days and nights. We categorized the patients who were successfully weaned off the ventilation into two groups: Group 1, patients who passed away in the hospital before discharge (general ward mortality patients), and Group 2, patients who were discharged from the hospital (discharged PMV patients). The group of patients who were unable to be successfully weaned off the ventilator included those who passed away in the RCC, those in hospice care in the RCC, and patients who still required ventilator support. Patients who still required ventilator support were transferred to the RCW (RCW patients).

### 2.4. Ethics Approval and Consent to Participate

The project received approval from the Research Ethics Committee of the Buddhist Dalin Tzu Chi Hospital (Approved IRB No.: B10802009).

### 2.5. Statistical Analysis

Differences in baseline characteristics, causes of respiratory failure, medical comorbidities, comorbidity numbers, and survival time were evaluated using Student’s t-test for continuous variables and Pearson’s chi-square or Fisher’s exact test for categorical variables. Sex, medical comorbidities, comorbidity numbers, RCC discharge status, and long-term survival of the five age cohorts were compared using a linear-by-linear association in the chi-square test. Of the 428 older patients on PMV, 117 were lost to follow-up. Finally, 311 older patients requiring PMV were included in the 1-year survival analysis. Univariate analysis was used to analyze the association of each variable with 1-year survival, and multivariate stepwise logistic regression models were used to assess the factors related to 1-year survival in the 311 older patients requiring PMV. Fisher’s exact test was used for combinations of two comorbidities between older prolonged mechanical ventilation patients who survived <1 year and patients who survived ≥1 year. We used the Kaplan–Meier method to estimate the cumulative probability of survival as a function of the number of months in the total number of older patients requiring PMV. The log-rank test was used to compare survival rates among the five age cohorts. The association between the 5-year survival rate between the four patient groups ((1) successfully weaned patients versus unsuccessfully weaned patients; (2) patients with tracheostomy versus patients without tracheostomy; (3) patients aged <75 years versus those aged ≥75 years; (4) patients with ≥ four comorbidities versus patients with < four comorbidities) was examined using the Cox proportional hazards model.

## 3. Results

Over the 6-year period, 574 patients were admitted to the RCC. Of these, 428 (74.6%) patients were aged ≥65 years, comprising 256 (59.8%) men and 172 (40.2%) women [3]. The demographic and clinical variables of the 574 patients requiring PMV used in the analyses are shown in Table 1. We found no difference in RCC discharge status, including successful weaning, ventilator dependence, RCC mortality, and general ward mortality, between patients aged <65 years and those aged ≥65 years. In terms of medical comorbidity, older patients requiring PMV had higher incidences of cardiovascular disease, chronic lung disease, chronic kidney disease, and metabolic disease than those aged <65 years. Older patients requiring PMV had less end-stage renal disease (ESRD) than those aged <65 years. In terms of the number of comorbidities, older patients requiring PMV showed higher incidences for three and four or more comorbidities than those aged <65 years. There were fewer older patients with no comorbidities requiring PMV than patients aged <65 years with no comorbidities requiring PMV. The percentage of patients requiring PMV who survived ≥1 year varied significantly between patients aged <65 years and those aged ≥65 years (39.1% vs. 19.9%, respectively; *p* < 0.001; odds ratio [OR] 0.387; 95% confidence interval [CI] 0.234–0.640). There was a notable disparity in the survival time between patients under 65 years old who needed PMV and those who were 65 years old or older (*p* < 0.001; mean survival time: 14.76 vs. 7.65 months, respectively).

In terms of 1-year survival, the univariate analysis of older patients requiring PMV (aged ≥ 65 years) revealed statistically significant differences in the following variables: age of <75 years, successful weaning, undergoing tracheostomy, and having four or more comorbidities between patients with <1-year survival and those with ≥1-year survival (Table 2). Multivariate analysis of older patients requiring PMV showed that those undergoing tracheostomy (*p* = 0.016; OR, 2.072; 95% CI, 1.143–3.758) and the successfully weaned patients (*p* = 0.017; OR, 2.158; 95% CI, 1.144–4.071) had better 1-year survival rates. Older patients requiring PMV with four or more comorbidities had poorer 1-year survival rates (*p* = 0.016; OR, 0.166; 95% CI, 0.039–0.714) (Table 3). Regarding comorbidity combinations between older prolonged mechanical ventilation patients who survived <1 year and patients who survived ≥1 year, there were statistically significant differences between the five combinations of two comorbidities between older prolonged mechanical ventilation patients who survived <1 year and patients who survived ≥1 year. The five combinations of two comorbidities are chronic kidney disease plus end-stage renal disease (*p* = 0.005), chronic kidney disease plus chronic liver disease (*p* = 0.021), chronic kidney disease plus metabolic disease (*p* = 0.034), underlying malignancy plus chronic kidney disease (*p* = 0.012), and underlying malignancy plus end-stage renal disease (*p* = 0.035). We classified patients who required PMV into 11 categories according to the cause of the acute respiratory failure that led patients to require PMV. The categories were: (1) pneumonia; (2) intracranial hemorrhage; (3) sepsis (not due to pneumonia); (4) postoperative condition; (5) COPD; (6) cardiac diseases; (7) underlying malignancies; (8) cervical spine diseases; and (9) miscellaneous causes. In terms of the cause of acute respiratory failure, there was no statistically significant difference between older patients requiring PMV who survived <1 year and those who survived ≥1 year (Table 2).

The demographic and clinical variables used in the analyses of older patients requiring PMV based on five age cohorts are shown in Table 4. There were no significant differences among the age cohorts in terms of sex, successful weaning, ventilator dependence, RCC mortality, and general ward mortality. Regarding medical comorbidity, the numbers of older patients requiring PMV with chronic lung disease, chronic kidney disease, and neurologic disease increased with age; however, the number of older patients requiring PMV with malignant disease and chronic liver disease decreased with age. In terms of the number of comorbidities, the number of older patients requiring PMV with four or more comorbidities increased with age, from 6.0% in the youngest cohort to 17.2% in the oldest cohort (*p* = 0.042).

The long-term follow-up data for the 311 older patients requiring PMV included those of cohorts aged 65–69 (*n* = 38), 70–74 (*n* = 49), 75–79 (*n* = 68), 80–84 (*n* = 85), and ≥85 (*n* = 71) years. The survival rates of all older patients requiring PMV decreased with age. The 1-year survival rate was 36.8% in the youngest cohort and 9.9% in the oldest cohort (*p* = 0.001). The 5-year survival rate was 10.5% in the youngest cohort and 0% in the oldest cohort (*p* < 0.001) (Table 4).

**Figure 1:**
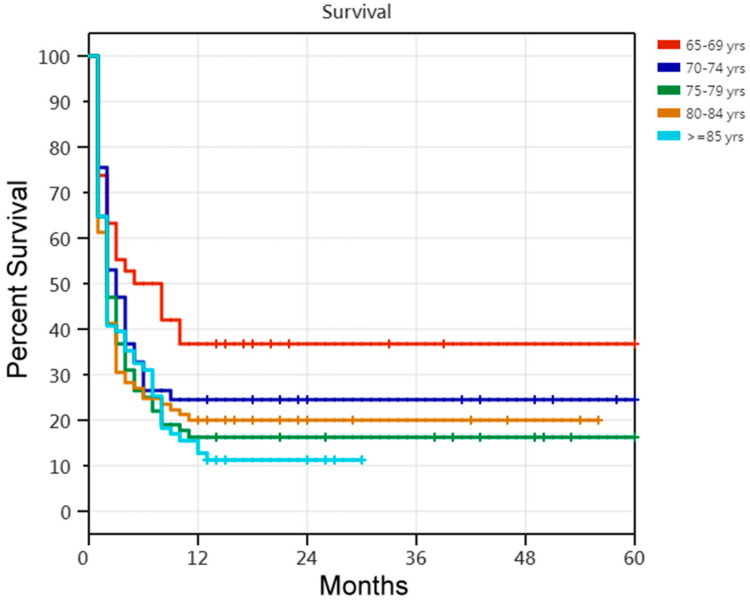
Kaplan–Meier survival curves for 311 older patients receiving prolonged mechanical ventilation in the five age cohorts. Figure 1 legend: Comparisons of five age cohorts of 311 older patients receiving prolonged mechanical ventilation (*p* = 0.048; log-rank test). Among the five age cohorts of older patients with PMV, there were age-related differences in the 5-year survival rate.

**Figure 2:**
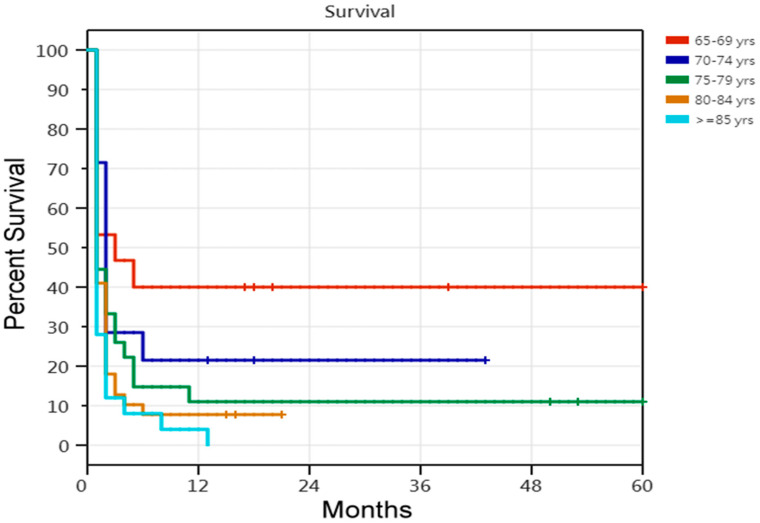
Kaplan–Meier survival curves of 121 unsuccessfully weaned older prolonged mechanical ventilation patients. Figure 2 legend: 1. Comparisons of five age cohorts of 121 unsuccessfully weaned older prolonged mechanical ventilation patients (*p* = 0.013; log-rank test). 2. According to Cox proportional-hazards regression analyses of the 121 unsuccessfully weaned older prolonged mechanical ventilation patients, the risk of death was 43% lower between those aged <75 and those aged ≥75 (*p* = 0.029; HR = 0.57; 95% CI 0.350–0.946).

**Figure 3:**
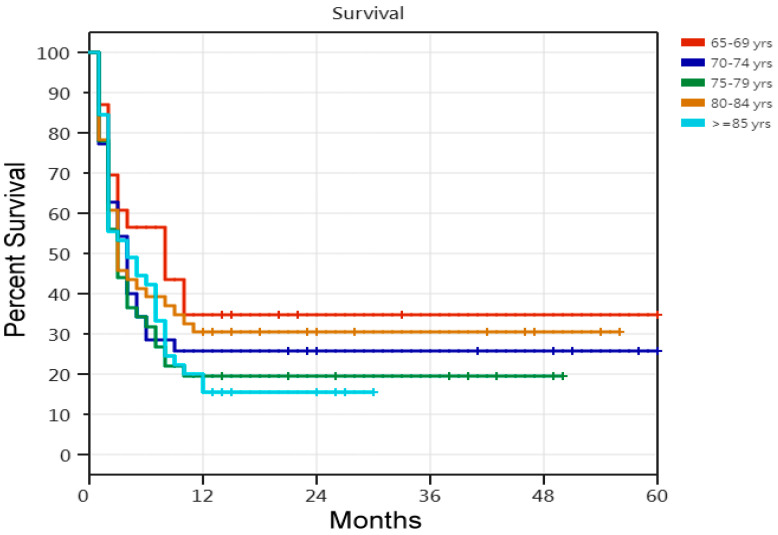
Kaplan–Meier survival curves of 190 successfully weaned older prolonged mechanical ventilation patients. Figure 3 legend: 1. Comparisons of five age cohorts of 190 successfully weaned older prolonged mechanical ventilation patients (*p* = 0.486; log-rank test). 2. The Cox proportional-hazards regression analyses of the 190 patients who were successfully weaned indicated that no factors were found to be associated with survival in this group.

Regarding the 5-year survival rate, according to the Cox proportional-hazards regression analysis of the 311 older PMV patients requiring PMV, the risk of death was 45% lower in the successfully weaned patients than in the unsuccessfully weaned patients. The risk of death was 46% lower in the patients undergoing tracheostomy than in those not undergoing tracheostomy. Older PMV patients with four or more comorbidities had an increased risk of death (Table 5). Comparisons of the Kaplan–Meier survival curves for the 311 older patients requiring PMV in the five age cohorts are shown in Figure 1. Among the five age cohorts of older patients with PMV, there were age-related differences in the 5-year survival rate. Comparisons of the Kaplan–Meier survival curves for the 121 unsuccessfully weaned older patients requiring PMV in the five age cohorts are shown in Figure 2. Cox proportional-hazards regression analysis of the 121 unsuccessfully weaned older patients requiring PMV showed that older patients requiring PMV aged <75 years showed a 43% reduction in death risk (HR, 0.57; 95% CI, 0.350–0.946; *p* = 0.029). Comparisons of the Kaplan–Meier survival curves for the 190 successfully weaned older patients requiring PMV in the five age cohorts are shown in Figure 3. Cox proportional-hazards regression analysis showed no relationship factors in the survival of the 190 successfully weaned older patients requiring PMV (*p* = 0.486; log-rank test).

## 4. Discussion

To the best of our knowledge, a limited number of studies have investigated the clinical outcomes of older patients requiring PMV. In a retrospective study, Frengley and colleagues investigated how advancing age affected the outcomes of 540 elderly individuals receiving prolonged mechanical ventilation in a public long-term acute care hospital. According to the study, the degree of respiratory physiology measure impairment in older persons with PMV, in addition to the burden of their concomitant illnesses, was a critical factor in determining the success of weaning and long-term survival. An additional benefit of successful weaning was a 62% decreased chance of mortality. While the likelihood of successfully weaning and achieving weaning requirements declined with age, age itself was not the primary predictor of outcomes [6]. Smolin B. et al. conducted an 18-month prospective observational cohort study involving all newly ventilated medical patients 65 years of age and older. This research was published during the peak of the COVID-19 epidemic, when intensive care unit services were at capacity. The number of elderly patients on ventilators in Israel has increased significantly more than the number of intensive care unit beds available. As a result, older ventilated patients received treatment in the internal medicine wards’ special units. Throughout the course of the trial, 554 individuals in all received mechanical ventilation. The hospital mortality rate was 64.1%. The 6-month survival rate was 26% overall. Thirty of the patients who made it through the hospital stay were still on prolonged mechanical ventilation, and 29 of the patients who were weaned off of it still had a tracheostomy. The greatest unfavorable predictors of survival after hospital discharge were a larger comorbidity burden, a low functional level before ventilation, and an age greater than 85 [7].

### 4.1. Clinical Variables between Patients Requiring PMV Aged <65 Years and Those Aged ≥65 Years

As age advances in older patients, their physiology progressively deteriorates because of the increasing burden of comorbidities. Comparing the clinical variables between patients requiring PMV aged <65 years and those aged ≥65 years, we found that the older PMV patients (aged ≥65 years) had more comorbidities such as cardiovascular disease, chronic lung disease, chronic kidney disease, and metabolic disease. Therefore, older patients requiring PMV showed higher incidences of comorbidities. Older patients requiring PMV showed lowed incidences of ESRD, which might be because of the poor long-term survival of these patients.

The RCC discharge status is the short-term outcome of patients requiring PMV, including successful weaning, RCC mortality, general ward mortality, and ventilator dependence. We found no significant differences in RCC discharge status between patients requiring PMV aged <65 years and those aged ≥65 years. From a long-term perspective, there was a significant difference in the 1-year survival and survival time between patients requiring PMV aged <65 years and those aged ≥65 years.

In terms of successful weaning, Su et al. demonstrated that patients requiring PMV aged ≥80 years have a lower rate of successful weaning and there was a trend towards successful weaning related to age group [8]. Lin et al. reported that the failed weaning rate showed no difference based on the patient’s age [9]. Cheng et al. showed that successful weaning from the mechanical ventilator is not related to age in patients requiring PMV [10]. Our previous data on patients requiring PMV revealed that age does not affect the weaning from the ventilator [3]. The present study confirmed that older patients requiring PMV have successful weaning rates similar to their younger counterparts.

Regarding long-term ventilator dependence, limited studies discuss long-term ventilator dependence in older patients requiring PMV. Su et al. showed that the group aged ≥65 years had more long-term ventilator-dependent patients than that aged <65 years [8]. However, the present study found that older patients requiring PMV had ventilator dependence similar to younger patients requiring PMV.

Very few studies have focused on age in relation to RCC mortality. Su et al. reported that RCC mortality is not associated with age [8]. Our previous study also showed that there is no association between increasing patient age and RCC mortality [3]. The present study confirmed that older patients requiring PMV have RCC mortality similar to younger patients requiring PMV.

### 4.2. Long-Term Survival Rate of Older Patients Requiring PMV

Regarding long-term survival, multiple reports have indicated that older patients in need of prolonged mechanical ventilation (PMV) have a significantly higher risk of poor survival [11,12,13,14]. Lai et al. demonstrated that age should not be the only determining factor for the outcome of very old patients requiring PMV [15]. However, Iregui et al. reported that increasing age is independently associated with hospital mortality in patients aged >60 years [16]. Frengley and colleagues demonstrated that age has minimal impact on long-term survival, with other clinical characteristics exerting a more significant influence on a patient’s survival. Patients have lower comorbidity burdens and better pulmonary physiology function, and these patients lead to a greater probability of successful weaning and long-term survival [6]. Our study revealed that age was not the only factor related to the long-term survival of older patients requiring PMV. The successfully weaned patients had better 1-year survival rates, which indicated better pulmonary physiology function. The results of the present study were similar to those reported in the abovementioned studies. Studies have shown that patients requiring PMV and who have had a tracheostomy have a higher chance of surviving for one year and a lower risk of in-hospital mortality [17,18,19]. The present study demonstrated that receipt or not of tracheostomy is also an influential factor in the long-term survival of older patients requiring PMV. In terms of the five combinations of two comorbidities, older patients who require prolonged mechanical ventilation and have two comorbidities had a poor 1-year survival rate. No studies explore the combination comorbidities related to prolonged mechanical ventilation. This study found that chronic the kidney disease comorbidity and the underlying malignancy comorbidity are the two key comorbidities of the five combinations of two comorbidities in patients who survived <1 year and patients who survived ≥1 year.

### 4.3. Clinical Features of Older Patients Requiring PMV Based on Five Age Cohorts

Regarding the short-term outcomes, our study revealed no significant age-related differences among the groups in successful weaning, ventilator dependence, RCC mortality and general ward mortality in older patients requiring PMV. Frengley et al. showed that the successful weaning rate progressively declines with increasing age. However, no independent age effect on successful weaning in older patients requiring PMV was observed in the multivariable analysis [6]. Iregui et al. showed no significant age-related differences in the successful weaning of older patients requiring PMV [16]. Su et al. also reported no significant age-related differences in successful weaning, RCC mortality, and ventilator dependence in patients requiring PMV aged ≥65 years [8]. The results of the present study were similar to those reported in the abovementioned studies.

### 4.4. Long-Term Outcomes of Older Patients Requiring PMV Based on Five Age Cohorts

Among older patients requiring PMV, age-related differences were noted in the 1-, 2-, 3-, 4-, and 5-year survival rates. Age was not the only factor affecting the long-term survival of older patients requiring PMV. In the unsuccessfully weaned older patients requiring PMV, there was an age-related difference among the groups in the 5-year survival rate in our study; age had a considerable effect on long-term survival. Older patients requiring PMV with poor physical respiratory function had unsuccessful weaning outcomes. This finding indicates that poor respiratory function combined with older age has a larger effect on survival. Our finding was different from that of Frengley et al., who reported no age-related differences in the 5-year survival of unsuccessfully weaned older patients requiring PMV [6]. Age in the successfully weaned older patients requiring PMV in our study showed no age-related differences among the groups in the 5-year survival rate. Successfully weaned older patients requiring PMV with satisfactory physical respiratory function had successful weaning outcomes, and therefore age had no significant effect on long-term survival. Our findings in this regard were similar to those reported by Frengley et al. [6]. The successful weaning of older patients requiring PMV is another important factor related to long-term survival.

The literature shows that patients requiring PMV who have undergone tracheostomy have a favorable 1-year survival rate and lower in-hospital mortality [14,15,16,17]. The present study demonstrated that the receipt or not of tracheostomy was an influential factor in the 1-year survival rate of older patients requiring PMV. Regarding the 5-year survival rate, according to the Cox proportional-hazards regression analysis of the 311 older PMV patients requiring PMV, the risk of death was 45% lower in the successfully weaned patients than in the unsuccessfully weaned patients. The risk of death was 46% lower in the patients undergoing tracheostomy than in those not undergoing tracheostomy. Older PMV patients with four or more comorbidities had an increased risk of death. Age was not the only influential factor related to the 5-year survival rate of older patients requiring PMV.

## 5. Limitations

We did not collect data on the patients’ nosocomial infection, nutritional status, oral and laryngo-pharyngeal health, laboratory findings, respiratory parameters, APACHE II score, Glasgow Coma Scale, or other variables related to the long-term outcomes of older patients requiring PMV. As a result, we could not establish a connection between any of these measures and the long-term outcomes of elderly patients requiring PMV. It is important to interpret our findings on these patients cautiously, as they are based on our retrospective study conducted in a single unit.

## 6. Conclusions

The age cohorts did not show a correlation with successful weaning, ventilator dependence, mortality in the RCC, or mortality in general wards among older patients requiring PMV. There were variations in the 5-year survival rate based on age among older patients undergoing PMV.

Age is not the sole determinant of long-term survival in elderly patients with PMV. Regarding the 5-year survival rate, the risk of death was 45% lower in the successfully weaned patients than in the unsuccessfully weaned patients. The risk of death was 46% lower in the patients undergoing tracheostomy than in those not undergoing tracheostomy. Successful weaning and receiving a tracheostomy are two other significant factors that impact long-term survival.

## Figures and Tables

**Table 1:** Comparison of clinical variables between patients aged <65 years and those aged ≥65 years (*N* = 574).

Variables	Age < 65 Years (*n* = 146)	Age ≥ 65 Years (*n* = 428)	*p*	OR
**RCC discharge status**				
Successful weaning	101 (69.2%)	290 (67.8%)	0.837	0.936
Ventilator dependence	22 (15.1%)	61 (14.3%)	0.787	0.937
RCC mortality	21 (14.4%)	74 (17.3%)	0.442	1.244
General ward mortality	15 (10.3%)	71 (16.6%)	0.080	1.737
**Medical comorbidity**				
Cardiovascular disease	59 (40.4%)	312 (72.9%)	<0.001	3.966
Chronic lung disease	10 (6.8%)	90 (21.0%)	<0.001	3.621
Chronic kidney disease	5 (3.4%)	57 (13.3%)	0.001	4.333
End-stage renal disease	17 (11.6%)	27 (6.3%)	0.047	0.511
Neurologic disease	25 (17.1%)	77 (18.0%)	0.900	1.062
Chronic liver disease	18 (12.3%)	31 (7.2%)	0.061	0.555
Metabolic disease	42 (28.8%)	163 (38.1%)	0.046	1.523
Malignant disease	25 (17.1%)	56 (13.1%)	0.270	0.729
**Number of comorbidities**				
None	32 (21.9%)	20 (4.7%)	<0.001	0.175
One	45 (30.8%)	107 (25.0%)	0.192	0.748
Two	44 (30.2%)	140 (32.7%)	0.608	1.227
Three	20 (13.7%)	112 (26.2%)	0.002	2.233
Four or more	5 (3.4%)	49 (11.4%)	0.003	3.646

**Table 2:** Comparison of clinical variables between respiratory care center patients aged ≥65 with 1-year survival (*N* = 311).

Variable	Survival < 1 Year (*n* = 249)	Survival ≥ 1 Year (*n* = 62)	*p*	OR
Successful weaning	144 (57.8%)	46 (74.2%)	0.020	2.096
Aged < 75 years	61 (24.5%)	26 (41.9%)	0.007	2.226
Tracheostomy	27 (10.8%)	18 (29.0%)	<0.001	3.364
**Causes of respiratory failure**				
Pneumonia	104 (41.8%)	23 (37.1%)	0.504	0.822
Intracranial hemorrhage	29 (11.6%)	9 (14.5%)	0.538	1.288
Sepsis	30 (12.0%)	6 (9.7%)	0.602	0.782
COPD	17 (6.8%)	6 (9.7%)	0.455	1.462
Cardiac diseases	18 (7.2%)	1 (1.6%)	0.133	0.210
Malignant diseases	12 (4.8%)	2 (3.2%)	0.591	0.658
Postoperative	18 (7.2%)	9 (14.5%)	0.074	2.179
Cervical spine diseases	2 (0.8%)	2 (3.2%)	0.161	4.117
Miscellaneous causes	19 (7.6%)	4 (6.5%)	0.751	0.835
**Medical comorbidity**				
Cardiovascular disease	181 (72.7%)	44 (71.0%)	0.874	0.981
Chronic lung disease	56 (22.5%)	15 (24.2%)	0.739	1.100
Chronic kidney disease	41 (16.5%)	4 (6.5%)	0.054	0.350
End-stage renal disease	24 (9.6%)	2 (3.2%)	0.126	0.313
Neurologic disease	92 (36.9%)	23 (37.1%)	1.000	1.066
Chronic liver disease	21 (8.4%)	3 (4.9%)	0.434	0.552
Metabolic disease	110 (44.2%)	23 (37.1%)	0.390	0.745
Malignant disease	40 (16.1%)	6 (9.7%)	0.236	0.560
**Number of comorbidities**				
None	9 (3.6%)	6 (9.7%)	0.088	2.857
One	58 (23.3%)	15 (24.2%)	0.868	1.051
Two	75 (30.1%)	20 (32.2%)	0.759	1.105
Three	67 (26.9%)	19 (30.6%)	0.634	1.200
Four or more	40 (16.1%)	2 (3.2%)	0.018	0.174

**Table 3:** The difference in clinical variables and tracheostomy between older prolonged mechanical ventilation patients who survived <1 year and those who survived ≥1 year.

	Odds Ratio	95% Confidence	*p* Value
**Univariate analysis**			
Successful weaning	2.096 (OR)	1.125–3.905	0.020
Age < 75 years	2.226 (OR)	1.245–3.980	0007
Tracheostomy	3.364 (OR)	1.707–6.629	<0.001
Four or more comorbidities	0.174 (OR)	0.041–0.742	0.018
**Multivariable analysis**			
Successful weaning	2.360 (OR)	1.225–4.548	0.010
Age < 75 years	1.700 (OR)	0.916–3.153	0.093
Tracheostomy	3.746 (OR)	1.785–7.864	<0.001
Four or more comorbidities	0.124 (OR)	0.028–0.557	0.006

**Table 4:** Clinical characteristics of older patients receiving prolonged mechanical ventilation based on five age cohorts (*N* = 428).

Characteristic	Age *					*p*
	65–69 (*n* = 50)	70–74 (*n* = 68)	75–79 (*n* = 101)	80–84 (*n* = 110)	≥85 (*n* = 99)	
**RCC discharge status**						
Successful weaning	33	50	70	68	69	0.722
Ventilator dependence	10	12	15	14	10	0.064
RCC mortality	7	7	15	26	19	0.070
General ward mortality	7	14	16	20	14	0.766
**Female**	15	24	46	43	44	0.122
**Medical comorbidity**						
Cardiovascular disease	33	48	77	83	71	0.479
Chronic lung disease	10	10	13	31	26	0.033
Chronic kidney disease	1	6	12	19	19	0.001
End-stage renal disease	5	5	5	9	3	0.196
Neurologic disease	4	20	44	56	40	<0.001
Metabolic disease	19	26	45	43	30	0.286
Malignant disease	15	9	11	12	9	0.003
Chronic liver disease	5	10	5	6	5	0.045
**Number of comorbidities**						
None	2	5	4	5	4	0.663
One	13	20	27	26	21	0.286
Two	26	20	31	33	30	0.053
Three	6	16	30	33	27	0.064
Four or more	3	7	9	13	17	0.042
**Length of survival** **†**						
1 year	14	11	11	15	7	0.001
2 years	6	7	9	6	3	0.014
3 years	5	7	8	5	0	0.001
4 years	4	4	4	2	0	0.002
5 years	4	1	1	0	0	<0.001

* Data are listed as numbers of patients. † The length of survival of 311 older prolonged mechanical ventilation patients included those of cohorts aged 65–69 (*n* = 38), 70–74 (*n* = 49), 75–79 (*n* = 68), 80–84 (*n* = 85), and ≥85 (*n* = 71) years. RCC, respiratory care center.

**Table 5:** The association between the 5-year survival rate between the four patient groups was examined using the Cox proportional hazards model.

	Hazard Ratio	95% Confidence	*p* Value
**Cox proportional-hazards model analysis**			
Successful weaning	0.546(HR)	0.422–0.707	<0.001
Age < 75 years	0.784(HR)	0.584–1.053	0.106
Tracheostomy	0.538(HR)	0.358–0.808	0.003
Four or more comorbidities	1.470(HR)	1.042–2.074	0.028

## Data Availability

The data produced and/or examined in the present study are not accessible to the public but can be obtained from the corresponding author upon request.
